# Clinical Characterization of Glaucoma in 63 Pet Rabbits (*Oryctolagus cuniculus*) in Japan

**DOI:** 10.1111/vop.70244

**Published:** 2026-08-20

**Authors:** Kumiko Kato, Makoto Nakata, Yasutsugu Miwa, Keiko Miyadera

**Affiliations:** ^1^ Department of Ophthalmology Kumi Animal Hospital Saitama Japan; ^2^ Japan Exotic Animal Medical Center Tokyo Japan; ^3^ Veterinary Medical Center, Graduate School of Agricultural and Life Sciences The University of Tokyo Tokyo Japan; ^4^ Department of Small Animal Clinical Sciences, College of Veterinary Medicine Michigan State University East Lansing Michigan USA

**Keywords:** gonioscopy, primary glaucoma, rabbit, secondary glaucoma

## Abstract

**Objective:**

To characterize the signalment and clinical presentation of glaucoma in pet rabbits.

**Animal Studied:**

Client‐owned pet rabbits (*n* = 222) referred to an exotic specialty animal hospital for ophthalmic examination between 2007 and 2023.

**Procedure:**

Medical records were retrospectively reviewed for rabbits diagnosed with glaucoma, to evaluate clinical findings and treatments. In addition to routine ophthalmic examination, gonioscopy and *Encephalitozoon cuniculi* serologic testing were performed when indicated.

**Results:**

Of 222 rabbits examined, 63 (28.4%) were diagnosed with glaucoma of which 33/63 (52.4%) were categorized as primary glaucoma and 27/63 (42.9%) as secondary glaucoma. Three other rabbits (4.8%) presented with unilateral secondary glaucoma with primary glaucoma risk in the fellow eye (mixed‐risk glaucoma). The mean age at diagnosis was 4.4 ± 2.8 years for primary and 10.0 ± 5.3 years for secondary glaucomas. Gonioscopy was performed in 23/33 (69.7%) of rabbits in the primary glaucoma group revealing a closed iridocorneal angle in 16/23 (69.6%). Median follow‐up was 707 days [IQR 126–1299] for primary and 59 days [IQR 1–1623] for secondary glaucomas. Medical management involved various topical antiglaucoma medications, with treatment regimens adjusted and often progressing to multi‐drug therapy. Bilateral glaucoma was ultimately documented in 75.8% (25/33) of primary and 29.6% (8/27) of secondary glaucomas. The most common underlying ocular condition associated with secondary glaucoma was suspected to be *E. cuniculi* infection.

**Conclusions:**

Both primary and secondary glaucomas are prevalent among pet rabbits in the Japanese population. Further studies are needed into the possible genetic factors underlying the development of glaucoma in rabbits.

## 1 Introduction

Glaucoma is a neurodegenerative disease affecting the retinal ganglion cells and the optic nerve, resulting in visual field loss and irreversible blindness. It is a common cause of blindness in veterinary species, with the most extensive clinical documentation in dogs [1, 2, 3]. In veterinary clinical settings, client‐owned pet rabbits are increasingly undergoing comprehensive ophthalmic examinations. However, the epidemiological and clinical features of glaucoma in this species remain poorly characterized. Glaucoma in pet rabbits is a clinically relevant yet underdefined condition, with limited evidence to guide diagnosis and management. This gap poses a challenge for veterinary practitioners and underscores the need for clinically grounded data on etiology, treatment, and outcomes.

Glaucoma can be categorized based on etiology: congenital glaucoma arises from developmental abnormalities and is typically present at or shortly after birth, primary glaucoma is typically associated with genetic factors, and secondary glaucoma results from ocular conditions, such as uveitis or neoplasia [4]. Spontaneous congenital glaucoma in rabbits was first described in 1886 by Schloesser [5]. However, research did not advance until the 1960s with the description of albino New Zealand rabbits spontaneously exhibiting congenital glaucoma and buphthalmia [6, 7, 8]. Buphthalmia in these rabbits was inherited as an autosomal recessive trait (*bu*) under semi‐lethal conditions, presenting bilaterally or unilaterally [9]. Bunt‐Milam et al. reported that newborns initially had normal intraocular pressure (IOP) (15–23 mmHg) that increased to 26–48 mmHg after 1–3 months. After 6–10 months, the eyes became buphthalmic and the IOP returned to normal or sub‐normal levels, consistent with congenital glaucoma [10]. Histopathological abnormalities included loss or compression of the iris pillars (i.e., pectinate ligaments) and posterior displacement or poor development of the aqueous plexus. Ultrastructural studies showed that the morphology of the glaucomatous trabecular meshwork was abnormal in all *bu*/*bu* rabbits by 2 weeks postnatally [11]. Others have reported replacement of the angular meshwork (i.e., the trabecular meshwork‐like structure in rabbits) with abundant extracellular matrix, basal lamina‐like material and unidentified round cells just beneath the aqueous plexus [12, 13]. However, these studies were limited to laboratory rabbits.

Glaucoma in pet rabbits has historically been reported mainly as sporadic occurrences [14]. More recently, retrospective studies have begun to provide insight into its clinical presentation and management. Yuschenkoff et al. reviewed 11 client‐owned rabbits (16 eyes) and found that many cases were not consistent with primary congenital glaucoma, suggesting a more heterogeneous etiology beyond the classic *bu/bu* model [15]. Topical therapy was effective in a number of cases, while refractory cases were successfully managed with enucleation or intravitreal gentamicin injection. In another study, the efficacy of intravitreal gentamicin injection was evaluated in blind eyes with end‐stage glaucoma in client‐owned rabbits [16]. Of the eight eyes (five rabbits) treated with intravitreal gentamicin, 87.5% had controlled IOP three months later with no overt complications. However, the types of glaucoma were not differentiated in those cases.

In a recent retrospective study, Damstén et al. evaluated 211 client‐owned rabbits and identified glaucoma in 35 rabbits [17]. Primary glaucoma was suspected in only four rabbits, and histopathology revealed inconsistent findings, including features of anterior segment dysgenesis in one case. The authors suggested that primary glaucoma in pet rabbits is underrecognized and highlighted the difficulty of achieving long‐term IOP control and vision preservation despite medical and surgical interventions.

Complementing these clinical observations, Li Puma et al. investigated the iridocorneal angle in healthy rabbits and demonstrated a correlation between gonioscopic grading and spectral‐domain optical coherence tomography (SD‐OCT) measurements [18]. This led to the development of a grading scheme for companion rabbits, supporting earlier identification of individuals at risk of glaucoma.

Despite these advances, existing studies are limited by small case numbers and heterogeneous case populations, underscoring the need for larger, clinically focused investigations to better define disease prevalence, etiology, and management outcomes in pet rabbits. In recent decades, with the increasing popularity of exotic pet animals in urban areas of Japan, demand for specialty clinical care has grown. The number of exotic pets requiring ophthalmic care has increased, with rabbits being the most common species presented for ocular disorders at the authors' specialty practice. Consequently, the authors have examined and managed a substantial cohort of pet rabbits with ocular conditions including glaucoma (Ohara et al. “A retrospective study of spontaneous ophthalmic diseases in companion rabbits ( *Oryctolagus cuniculus* ): 297 eyes from 205 rabbits (2007–2022)” *under revision*). In this study, we aimed to provide real‐world data through a long‐term retrospective investigation of a larger cohort of pet rabbits in Japan to determine the prevalence of primary and secondary glaucomas, identify contributing factors, and describe long‐term therapeutic outcomes.

## 2 Materials and Methods

### 2.1 Animals

Included in this study for evaluation were client‐owned pet rabbits that presented between 2007 and 2023 to Japan Exotic Animal Medical Center, an exotic animal hospital. This institution is a referral exotics specialty hospital that serves the densely populated Tokyo region in Japan where small exotic animals such as rabbits are popular due to compact urban living conditions, themselves colloquially referred to as “rabbit hutch”. The animals presented to this exotic animal hospital for ocular problems were evaluated by a board‐certified veterinary ophthalmologist (KK). Medical records for all the animals affected by glaucoma were reviewed to derive data from the initial presentation and all the subsequent follow‐up visits. Clinical data extracted included ophthalmic examination findings and therapeutic regimen.

### 2.2 Ophthalmic Examination

All ocular examinations were performed with the rabbits under gentle manual restraint, without sedation. A complete ophthalmic examination was performed including tonometry using a handheld applanation tonometer (Tono‐Pen Vet, Reichert), slit‐lamp biomicroscopy (SL‐15, Kowa), as well as indirect ophthalmoscopy (IO‐αTV, Neitz) using a 30D double aspheric lens (VOLK, R E Medical). In general, an IOP > 25 mmHg was used as the diagnostic criterion for glaucoma. However, clinical history and timing of presentation were also taken into account, such as normotensive but buphthalmic eye indicating prior IOP elevation. In some cases, IOP values mildly above 25 mmHg without clinical evidence of glaucoma were thought to possibly reflect immobilization stress [19], and the disease status was comprehensively determined by the clinician. Gonioscopy was performed when indicated using a 17‐mm Koeppe gonio lens (Ocular Instruments) in the affected and/or the fellow eye, as permitted by corneal clarity. Fundus photographs were taken using a fundus camera (VersaCam α, NIDEK). Tonometry and gonioscopy were performed after application of topical anesthetic drops (0.4% oxybuprocaine hydrochloride, Santen Pharmaceutical) to the ocular surface. One minute later, the IOP was measured, followed by gonioscopy using a hydroxyethylcellulose‐based coupling agent (Scopizol, Senju Pharmaceutical).

All examinations and measurements were performed in the clinically unaffected eye first, followed by the eye presenting with problems. Each eye was evaluated as either normal or diseased by the same board‐certified veterinary ophthalmologist (KK). In cases of glaucoma, the type of glaucoma was mainly determined based on the presence or absence of underlying ocular conditions. Gonioscopy was performed in selected cases, further informing the classification of the glaucoma types. Serum *Encephalitozoon cuniculi* (*E. cuniculi*) antibody titers were determined by a commercial diagnostic laboratory (FUJIFILM VET Systems) using an indirect fluorescent antibody test. Titers of ≥ 1:80 to *E. cuniculi* antigens were considered positive according to the test provider.

### 2.3 Statistical Analyses

All data analyses were carried out using Prism (GraphPad), Statcel 3 (OMS Publishing Inc.), or Microsoft Office Excel. The Mann–Whitney *U* test was used to analyze differences in sex, age of onset, affected eye, and duration of follow‐up between primary and secondary glaucomatous rabbits. The onset of glaucoma and the affected eye between glaucoma types were analyzed using two‐factor analysis of variance and the Kruskal–Wallis test. The onset of glaucoma in each breed of rabbits was analyzed using the chi‐square test for independence 2 × 2 contingency tables. The chi‐square test for independence *m* × *n* contingency table was analyzed between glaucoma types and the affected eye, sex and glaucoma types, and breeds and glaucoma types. The expectancy of glaucoma onset according to age and sex in the primary and secondary glaucomatous groups was analyzed using simple regression analysis. The level of significance was set at *p* < 0.05.

## 3 Results

### 3.1 Study Population

Two hundred and twenty‐two pet rabbits (131 male, 91 female) were included in this study. These rabbits were subjected to a complete ophthalmic examination to assess ocular abnormalities. Thirteen breeds of rabbits were represented, where Holland Lop (*n* = 76; 44 male, 32 female) and Netherland Dwarf (*n* = 73; 40 male, 33 female) were by far the most common breeds examined (Table 1). Of the 222 rabbits examined, 63 (28.4%), representing eight breeds and unknown breeds, were diagnosed with glaucoma. Of the glaucoma cases, 52.4% (n=33) had primary and 42.9% (n=27) had secondary glaucomas. In addition, mixed‐risk glaucoma in which there was one eye with primary glaucoma and the other eye with secondary glaucoma, was detected in 4.8% (n=3) of the pet rabbits affected by glaucoma. A complete list of animals diagnosed with primary, secondary, and mixed‐risk glaucomas is presented in Tables 2, 3, 4, respectively.

**TABLE 1:** Rabbit breeds examined and glaucoma cases by breed.

Breeds	Animals examined (*n*)	All glaucoma cases (*n*)	All glaucoma prevalence (%)	Primary glaucoma cases (*n*)	Secondary glaucoma cases (*n*)	Mixed‐risk glaucoma cases (*n*)
Holland Lop	76	31	40.8	18	12	1
Netherland Dwarf	73	15	20.5	6	7	2
Lion Head	7	1	14.3	1	—	—
American Fuzzy Lop	4	0	0.0	—	—	—
Jersey Wooly	4	1	25.0	1	—	—
Mini Rex	4	2	50.0	1	1	—
Dutch‐rabbit	2	1	50.0	1	—	—
Dwarf Hotot	2	1	50.0	—	1	—
Lop Ear	2	0	0.0	—	—	—
Angora‐rabbit	1	0	0.0	—	—	—
Himalayan‐rabbit	1	1	100.0	1	—	—
Mini Lop	1	0	0.0	—	—	—
Rex‐rabbit	1	0	0.0	—	—	—
Mix	31	7	22.6	3	4	—
Unknown	13	3	23.1	1	2	—
Total	222	63	28.4	33	27	3

*Note:* *n*, number of rabbits.

### 3.2 Primary Glaucoma

**FIGURE 1:**
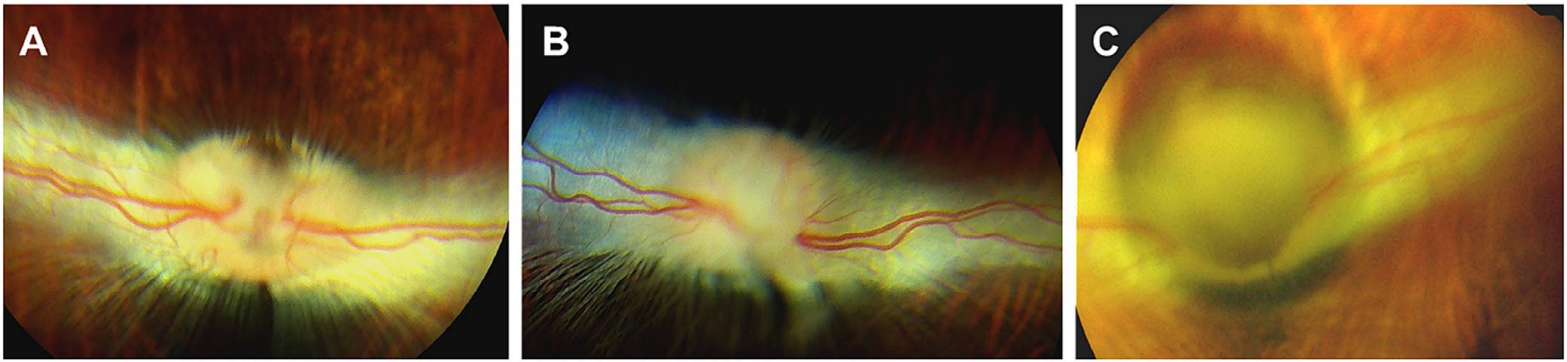
Fundus photographs of pet rabbits. Optic disc appearance in (A) a normal rabbit (case #44, left eye); (B) an early‐stage glaucomatous eye with preserved dazzle reflex and focal optic disc cupping (case #10, right eye); and (C) an advanced glaucomatous eye that is blind, showing complete optic disc cupping (case #8, left eye).

There were 33 rabbits categorized as primary glaucoma. The diagnosis was based on the lack of concurrent ocular or systemic abnormalities that could lead to glaucoma while exhibiting increased IOP (> 25 mmHg), historically documented or clinical evidence of historically increased IOP such as optic disc cupping (Figure 1), buphthalmia, and phthisis bulbi (Table 2). Of the affected rabbits, 17 were diagnosed bilaterally at initial presentation, typically with evidence of asymmetry in historical disease onset and progression. Additional 8 rabbits, though initially affected in one eye, later developed glaucoma in the fellow eye thus ultimately 75.8% (25/33) of rabbits in the primary glaucoma group being affected bilaterally. Included in this group are two rabbits (Cases #10 and #33) that were classified based on abnormal gonioscopic findings bilaterally and initiated on prophylactic treatment without developing clinical glaucoma during the follow‐up period.

**TABLE 2:** Signalment and initial findings of rabbits diagnosed with primary glaucoma

Case #	Breed	Sex	Diagnosis age (years)	Affected Eye	IOP		Gonioscopy	*E. cuniculi* titer	Clinical findings at diagnosis	Initial treatment	Follow‐up (days)
					R	L					
#1	Mix	M	0.6	OU	44	40	—	—	OU: Buphthalmos, corneal edema, no evidence of intraocular inflammation/tumor	Xalatan BID, Hypadil TID (OU)	1224
#2	Unknown	M	3.9	OD (→OU)	45	19	OS: Closed	—	OD: Buphthalmos (6‐month duration), diagnosed glaucoma by another vet ophthalmologist 6 months before presentation	Xalatan BID, Hypadil TID (OD); Hypadil BID (OS*)	1505
#3	Netherland Dwarf	M	6.9	OD (→OU)	30	23	OS: Closed‐Narrow	—	—	Xalatan BID, Hypadil BID (OD); Trusopt BID (OS*)	1176
#4	Holland Lop	NF	8.3	OD	18	14	OS: Closed	—	OD: Buphthalmos, corneal edema	Hypadil BID (OU [OS*])	1
#5	Holland Lop	M	7.2	OU	39	41	OD: Closed	—	OS: Anterior lens subluxation	Trusopt TID (OU)	147
#6	Himalayan‐Rabbit	NM	6.4	OU	38	24	OD: Closed	—	OD: Optic disk enlargement/cupping; OS: Mild buphthalmos, corneal edema/erosion	Xalatan BID, Hypadil BID (OU)	2583
#7	Holland Lop	M	8.7	OU	24	39	—	—	OU: No intraocular inflammation, peripheral corneal clouding	Xalatan BID, Hypadil TID (OU)	1345
#8	Mix	M	10.7	OS (→OU)	15	34	OS: Narrow	—	OD: Indolent corneal ulcer; OU: optic disk cupping	Xalatan BID, Hypadil TID (OU)	112
#9	Netherland Dwarf	F	2.1	OU	26	27	—	—	OU: Buphthalmos, corneal edema, KCS	Xalatan BID, Hypadil TID (OU)	700
#10	Dutch‐Rabbit	M	2.8	OU	25	25	OU: Narrow	—	Normal eye exam. Littermate developed glaucoma.	Hypadil SID (OU)	990
#11	Holland Lop	NF	6.6	OU	50	23	OS: Narrow	—	—	Glanatec BID (OD), SID (OS*)	706
#12	Mini Rex	NM	5.3	OU	41	16	OS: Closed	—	OD: Buphthalmos, corneal edema, peripheral anterior synechiae	Travatanz BID, Hypadil TID (OD)	1879
#13	Lion Head	M	7.9	OU	35	34	—	—	OU: Buphthalmos, peripheral corneal degeneration	Glanatec TID, Xalatan BID, Hypadil TID (OU)	391
#14	Netherland Dwarf	F	4.8	OD (→OU)	20	25	OS: Closed	—	OD: Slight buphthalmos	Glanatec BID, Trusopt BID (OD); Glanatec BID (OS*)	1
#15	Holland Lop	F	8.7	OD	34	17	OS: Closed	—	—	Cosopt TID (OD), BID (OS)	126
#16	Holland Lop	NM	7.5	OD (→OU)	48	24	OS: Closed	—	OD: Buphthalmos, corneal clouding	Glanatec TID, Trusopt TID (OD); Glanatec BID, Trusopt SID (OS*)	1281
#17	Holland Lop	M	3.2	OS (→OU)	14	33	OD: Narrow‐Closed	—	OD: Shallow anterior chamber; OS: Buphthalmos, corneal edema	Glanatec BID (OD*); Glanatec TID + Trusopt TID (OS)	574
#18	Jersey Wooly	NF	5.8	(OD→) OU	Enucleated	47	OS: Closed	—	OS: Lagophthalmos, chronic corneal erosion/scarring, suspected eosinophilic keratopathy; OD: historically enucleated due to corneal perforation	Glanatec TID, Rescula TID, Cosopt TID (OS)	721
#19	Holland Lop	NM	6.4	OU	30	38	OD: Closed	—	—	Xalatan BID + Hypadil TID (OS)	1099
#20	Holland Lop	NF	6.4	OU	35	31	—	—	OU: Buphthalmos, corneal edema, staphyloma	Glanatec TID, Cosopt TID (OU)	1
#21	Holland Lop	F	7.4	OS (→OU)	13	42	OD: Closed	—	OS: Indolent corneal ulcer	Glanatec BID (OD*); Glanatec TID, AzorgaTID (OS)	1372
#22	Holland Lop	NF	5.2	OU	45	37	—	—	OU: Buphthalmos, no evidence of inflammation	Rescula TID, Cosopt TID (OU)	1579
#23	Holland Lop	NM	5.7	OU	45	30	—	—	OU: Buphthalmos, mild staphyloma, peripheral anterior synechiae	Cosopt TID (OD); BID (OS)	27
#24	Holland Lop	F	3.4	OD (→OU)	58	19	OS: Closed	1:80	OD: Buphthalmos, corneal degeneration	Rescula TID, Cosopt TID (OD), Cosopt BID (OS)	514
#25	Holland Lop	F	2.8	OU	40	36	—	—	OU: Buphthalmos, staphyloma, peripheral corneal degeneration	Glanatec BID (OU)	707
#26	Holland Lop	M	7.6	OU	26	31	OU: Closed	—		Glanatec BID (OU)	1365
#27	Holland Lop	NM	7.8	OD	30	20	—	—	OD: Facial nerve paralysis (lower lid) secondary to ipsilateral otitis media	NT (Supportive therapy for facial nerve paralysis was prioritized)	1
#28	Holland Lop	M	8.3	OD	30	25	OS: Closed	—	—	Glanatec TID (OD), BID (OS)	1204
#29	Holland Lop	M	9.4	OD	36	23	OS: Closed	—	OD: Buphthalmos, peripheral corneal degeneration	Glanatec TID, Cosopt TID (OD); Cosopt BID (OS)	896
#30	Netherland Dwarf	M	3.3	OS	17	24	OD: Narrow	—	OS: Blind, mydriasis	Glanatec BID (OU)	282
#31	Netherland Dwarf	NF	8.7	OU	15	13	—	1:160^†^	OU: Buphthalmos, posterior lens subluxation, no intraocular inflammation or abscess.	Azopt BID (OU)	252
#32	Holland Lop	M	7.2	OS	30	45	OD: Closed	—	OS: Buphthalmos	Glanatec BID, Hypadil BID (OD); Glanatec TID, Rescula TID (OS)	357
#33	Mix	M	7.9	OU	16	14	OD: Narrow‐Slightly narrow, OS: Narrow	—	Seeking preventing care as a sibling bunny developed glaucoma	Glanatec SID (OU*)	980

*Note:* Drugs (brand names are bolded; see Table 5 for concentrations (%)): **Aiphagan**, Brimonidine; **Azorga**, Brinzolamide + Timolol; **Azopt**, Brinzolamide; **Cosopt**, Dorzolamide + Timolol; **Detantol**, Bunazosin; **Eybelis**, Omidenepag; **Glanatec**, Ripasudil; **Hypadil**, Nipradilol; **Mirol**, Levobunolol; **Rescula**, Isopropyl Unoprostone; **Tapros**, Tafluprost; **Timoptol**, Timolol; **Travatanz**, Travoprost; **Xalatan**, Latanoprost. **‐**, not performed

Abbreviations: **BID**, twice daily; ***E. cuniculi***, *Encephalitozoon cuniculi*; **F**, female; **NF**, neutered female; **NM**, neutered male; **NT**, no treatment; **OD**, right eye; **OS**, left eye; **OU**, both eyes; **PO**, by mouth; **SID**, once daily; **TID**, three times daily; **TL**, too low to measure.

* Prophylaxis.

† Historical *E. cuniculi* infection 1.5 years prior to ocular presentation, when neurologic signs (torticollis and body torsion) were the only findings.

Specifically, the absence of current or historical intraocular inflammation, intraocular tumor, and intraocular abscess were determined. The affected eye(s) was found to be buphthalmic in 18 animals at the time of initial presentation. Gonioscopy was performed in 23/33 (69.7%) of the rabbits in the primary glaucoma group, primarily tested in the contralateral normal eye. The finding of abnormal iridocorneal angle (i.e., closed or narrow) was part of the criteria for the diagnosis of primary glaucoma (refer to Gonioscopy subsection). Gonioscopy could not be performed in 10/33 rabbits (30.3%) due to factors including corneal edema, severe buphthalmia, and peripheral corneal degeneration, precluding visualization of the iridocorneal angle.

### 3.3 Secondary Glaucoma

Secondary glaucoma was diagnosed in 27 rabbits based on the presence of concurrent or historic ocular or systemic abnormalities that could lead to glaucoma while exhibiting increased IOP (> 25 mmHg), historically documented, or clinical evidence of historically increased IOP (Table 3). Diffuse corneal edema was a common finding while 14 rabbits exhibited unilateral or bilateral buphthalmia. Of the rabbits in this disease subtype, 29.6% (8/27) were affected bilaterally.

**TABLE 3:** Signalment and initial findings of rabbits diagnosed as secondary glaucoma

Case #	Breed	Sex	Diagnosis age (years)	Affected eye	IOP		Gonio scopy	*E. cuniculi* titer	Underlying findings	Clinical findings at diagnosis	Initial treatment	Follow‐up (days)
					R	L						
#34	Holland Lop	NF	8.0	OU	10	34	—	—	Cataract/ Uveitis	OU: Mature cataract, KPs, anterior synechiae; OD: Corneal degeneration, iris cyst. OS: Buphthalmos, corneal edema, posterior synechiae	Xalatan BID, Hypadil BID (OS)	896
#35	Unknown	F	0.8	OS	29	14	—	1:<80 (seronegative)	Uveitis (Hx)	OS: Mild buphthalmos, corneal edema, recurrent corneal ulcers; evidence of posterior synechia, focal anterior cortical cataract; [age 2y] Enucleation due to buphthalmos and corneal perforation	NT (borderline IOP)	1
#36	Holland Lop	F	7.1	OD	30	10	—	—	Trauma	OD: History of traumatic ocular laceration, secondary cataracts, iris bombe, and secondary glaucoma; anterior lens luxation, buphthalmos, staphyloma, posterior synechiae	NT (difficulty medicating)	1
#37	Mix	M	0.8	OS	20	14	—	1:40	Uveitis (Hx)	OS: Posterior synechiae, cataracts, shallow anterior chamber, susp. *E. cuniculi*; [14 months later] Phthisis bulbi	Flumetholon BID, Ofloxacin BID (OS)	802
#38	Holland Lop	M	8.3	OS	20	35	—	—	Uveitis	OS: Buphthalmos, corneal edema, chronic corneal ulcer, anterior uveitis	Hypadil SID (OD*); TID (OS)	1
#39	Mini Rex	F	6.3	OS	12	35	—	1:40	Uveitis	OS: Buphthalmos, corneal edema, chronic corneal ulcer, anterior uveitis	Trusopt TID (OS)	2779
#40	Dwarf Hotot	M	0.5	OS	20	20	—	1:40	*E.cuniculi* (susp.)	OS: Intraocular abscess between iris and lens (susp. *E. cuniculi*)	Trusopt BID (OS)	105
#41	Netherland Dwarf	M	0.8	OD	18	20	OS: Open	1:160	*E. cuniculi*	OD: Intraocular abscess, posterior synechiae	NT (normotensive)	1692
#42	Netherland Dwarf	M	1.4	OD	50	17	—	—	*E.cuniculi* (susp.)	OD: Intraocular abscess (susp. *E. cuniculi*), posterior synechiae, corneal edema	Cosopt BID (OD)	3351
#43	Netherland Dwarf	F	1.0	OU	27	28	—	1:160	*E. cuniculi*	OD: Corneal ulcer; OS: Facial nerve paralysis due to trauma from uncontrolled body posture (circling)	Trusopt BID (OU)	325
#44	Unknown	M	8.9	OD	26	19	—	1:40	*E.cuniculi* (susp.)	OD: Corneal stromal abscess (susp. *E. cuniculi*), iritis	Trusopt BID (OD)	1148
#45	Netherland Dwarf	M	1.3	OD	10	20	—	1:160	*E. cuniculi*	OD: Intraocular abscess, pigment deposition on the anterior lens capsule; OU: KPs	Curcuminoid, Fenbendazole, Antibiotics PO	3811
#46	Mix	F	11.5	OD	27	15	—	—	Mass/ Uveitis	OD: Peripheral iris mass with scleral pigmentation (melanoma susp.); enucleation and histopathology revealed inflammation not tumor	NT (enucleation, OD)	1
#47	Holland Lop	NM	7.6	OD (→OU)	31	15	—	1:40	Uveitis	OD: Buphthalmos, KPs, posterior synechiae; OS: Anterior synechiae secondary to corneal perforation	Travatanz BID, Hypadil TID (OD); Trusopt BID (OS)	56
#48	Mix	F	13.1	OD	18	10	—	—	Uveitis (Hx)	OD: Mild buphthalmos, lagophthalmos, peripheral corneal vascularization, superior iris congestion, posterior synechiae, lens subluxation	Glanatec TID (OD)	218
#49	Holland Lop	F	6.2	OU	23	30	—	—	Uveitis	OU: Anterior uveitis; pericardial effusion	Glanatec TID, Cosopt TID (OU)	199
#50	Holland Lop	M	4.8	OU	18	19	OD: Open	1:160	*E. cuniculi*/ Cataract	OU: Mature cataract, focal peripheral anterior synechiae; OS: Shallow anterior chamber with lens subluxation.	Trusopt BID (OS)	2364
#51	Holland Lop	F	5.8	OU	20	20	—	1:160	*E. cuniculi*	OU: Buphthalmos; [age 4y] Developed vestibulopathy	NT (normotensive)	1
#52	Netherland Dwarf	M	0.5	OS	15	50	—	—	Perforation	OS: Buphthalmos secondary to corneal perforation	NT (difficulty medicating)	1
#53	Netherland Dwarf	F	9.9	OU	15	23	—	—	Uveitis (Hx)	OU: Corneal dystrophy/degeneration, posterior synechia; OS: Mild buphthalmos	NT (normotensive)	56
#54	Netherland Dwarf	M	6.8	OD	TL	24	—	1:160	*E. cuniculi*	OD: Phthisis bulbi, intraocular abscess	NT (phthisis bulbi)	1
#55	Holland Lop	M	6.4	OU	55	45	—	—	Cataract/ Uveitis	OU: Buphthalmos, corneal edema, mature cataract with associated peripheral anterior synechiae	Travatanz BID, Hypadil TID, Trusopt TID (OU)	147
#56	Holland Lop	NM	4.1	OD	23	20	—	—	*E.cuniculi* (susp.)	OD: Intraocular abscess (susp. *E. cuniculi*)	NT (normotensive)	1
#57	Holland Lop	M	3.0	OS	28	28	—	—	Trauma	OS: Traumatic staphyloma, secondary mild buphthalmos	NT (borderline IOP)	1
#58	Holland Lop	NF	7.6	OS	8	38	—	—	Uveitis (Hx)	OS: Mild buphthalmos, partial peripheral anterior synechiae, clot on the posterior lens capsule	Glanatec TID, Trusopt TID (OS)	59
#59	Mix	M	10.6	OS	18	30	—	—	Mass	OS: Buphthalmos, hyphema, posterior synechiae, mild dark iris discoloration, iris vascularization, small mass over anterior lens capsule (8‐9 o’clock); possible ciliary body tumor	Hypadil TID, Trusopt TID (OS)	1
#60	Netherland Dwarf	M	12.4	OD	47	17	OS: Open	1:160	*E. cuniculi*/ Cataract	OD: Uveitis, recurrent corneal ulcers with scarring; OU: Mature cataract, horizontal nystagmus	Timoptol TID (OD)	57

*Note:* Drugs (brand names are bolded; see Table 5 for concentrations (%)): **Aiphagan**, Brimonidine; **Azorga**, Brinzolamide + Timolol; **Azopt**, Brinzolamide; **Cosopt**, Dorzolamide + Timolol; **Detantol**, Bunazosin; **Eybelis**, Omidenepag; **Glanatec**, Ripasudil; **Hypadil**, Nipradilol; **Mirol**, Levobunolol; **Rescula**, Isopropyl Unoprostone; **Tapros**, Tafluprost; **Timoptol**, Timolol; **Travatanz**, Travoprost; **Xalatan**, Latanoprost. **‐**, not performed;

Abbreviations: **BID**, twice daily; ***E. cuniculi***, *Encephalitozoon cuniculi*; **F**, female; **Hx**, history; **KP**, keratic precipitate; **NF**, neutered female; **NM**, neutered male; **NT**, no treatment; **OD**, right eye; **OS**, left eye; **OU**, both eyes; **PO**, by mouth; **SID**, once daily; **susp**, suspected; **TID**, three times daily; **TL**, too low to measure.

* Prophylaxis.

The most common concurrent abnormal finding was anterior uveitis of various etiology. Of these, *E. cuniculi* infection was most frequently found as indicated by the characteristic intraocular abscesses associated with the lens, iris, or corneal extension, identified in at least seven rabbits. *E. cuniculi* titers were performed in 13 rabbits in the secondary glaucoma group, ranging from 1:40 (*n* = 5) and 1:< 80 (*n* = 1) to 1:160 (*n* = 7). Lower titers were observed in rabbits with intraocular abscesses (*n* = 2) or in those without abscesses but with evidence of prior anterior uveitis (*n* = 3), while higher titers (1:160) were also identified in rabbits with intraocular abscesses (*n* = 3). Additionally, four rabbits presented with bilateral mature cataracts; of the two tested, both had the higher *E. cuniculi* titer of 1:160. Overall, 11 rabbits (9 males, 2 females) were determined to have *E. cuniculi* as the underlying findings of secondary glaucoma, based on *E. cuniculi* titers and/or the presence of characteristic intraocular abscesses. Peripheral anterior synechiae were common findings in eyes with mature cataracts suggesting an etiology of either intumescent lens changes resulting in collapse of the iridocorneal angle, possible *E. cuniculi* infection, and/or lens‐induced uveitis, underlying the development of secondary glaucoma. Four rabbits in the secondary glaucoma group had gonioscopy performed, all of which had open iridocorneal angle.

Non‐specific clinical findings indicative of historical anterior uveitis included keratic precipitates and posterior synechiae. Other underlying findings were ocular trauma (*n* = 2) and intraocular mass (*n* = 2). Historical corneal perforation was found in three rabbits in the secondary glaucoma group; one perforation was thought to be the original cause of secondary glaucoma, likely as a sequel of uveitis, while the etiology of the other two perforations was unclear.

### 3.4 Mixed‐Risk Glaucoma

Three rabbits were classified as “mixed‐risk glaucoma”, here referring to a clinical presentation in which one eye has secondary glaucoma while the fellow eye is gonioscopically at risk for primary glaucoma (Table 4).

**TABLE 4:** Signalment and initial findings of rabbits diagnosed as mixed‐risk glaucoma

Case #	Breed	Sex	Diagnosis age (years)	Affected eye	IOP		Gonioscopy	*E. cuniculi* titer	Clinical findings at diagnosis	Initial treatment	Follow‐up (days)
					R	L					
#61	Netherland Dwarf	M	4.9	OD primary; OS secondary	24	29	OD: Closed (5.0y)	1:160	OS: Intraocular abscess between lens and iris, contacting the corneal endothelium, with posterior synechiae.	Fenbendazole, Enrofloxacin, Sulfamethxazole, Trimetoprim PO	2128
#62	Holland Lop	M	7.4	OD primary; OS secondary	16	38	OD: Narrow to closed	1:320	OS: Mild buphthalmos, posterior synechiae, peripheral anterior synechiae, suspected to be secondary to prior intraocular inflammation.	Glanatec BID (OD*); Glanatec TID, Cosopt BID (OS)	973
#63	Netherland Dwarf	M	6.2	OD secondary; OS primary	TL	29	OS: Closed	1:40	OD: Intraocular inflammation and abscess present since *age 1.5y*, with *E.cuniculi* infection suspected. The eye has progressed to phthisis bulbi.	Glanatec BID (OS)	322

*Note:* Drugs (brand names are bolded; see Table 5 for concentrations (%)): **Cosopt**, Dorzolamide + Timolol; **Glanatec**, Ripasudil.

Abbreviation: **BID**, twice daily; ***E. cuniculi***, *Encephalitozoon cuniculi*; **OD**, right eye; **OS**, left eye; **PO**, by mouth; **TID**, three times daily; **TL**, too low to measure.

* Prophylaxis.

*Case #61* (Netherland Dwarf, male, 4.9 years): The left eye (IOP 29 mmHg) was diagnosed with secondary glaucoma based on an intraocular abscess, posterior synechiae, and an elevated *E. cuniculi* titer (1:160). Systemic treatment (fenbendazole, enrofloxacin, sulfamethoxazole‐trimethoprim) was initiated (Table S3). Topical ripasudil was started in the left eye but discontinued due to phthisis bulbi. The right eye was later started on ripasudil following identification of a closed angle on gonioscopy; despite IOP increasing up to 27 mmHg, vision was maintained through 82 months post initial encounter in this second eye.

*Case #62* (Holland Lop, male, 7.4 years): Secondary glaucoma was diagnosed in the buphthalmic left eye (IOP 38 mmHg), with evidence of prior inflammation (posterior and peripheral anterior synechiae) and an elevated *E. cuniculi* titer (1:320). The right eye was clinically normal (IOP 16 mmHg) but had a narrow‐to‐closed angle on gonioscopy. The left eye was treated with ripasudil and dorzolamide/timolol, and prophylactic ripasudil was initiated in the right eye. By 35 months post encounter, IOP in the right eye increased to 42 mmHg without identifiable underlying disease, meeting the criteria of primary glaucoma.

*Case #63* (Netherland Dwarf, male, 6.2 years): The left eye (IOP 29 mmHg) had primary glaucoma confirmed by a closed angle on gonioscopy and was treated with ripasudil. The right eye had already exhibited phthisis bulbi with chronic uveitis and abscessation since 1.5 years of age consistent with prior secondary glaucoma. Although a titer of 1:40 was not confirmatory, characteristic clinical findings were deemed consistent with *E. cuniculi* infection.

Given the genetic basis of primary glaucoma, both eyes are inherently predisposed to developing the disease. These cases demonstrate that secondary glaucoma may occur in eyes already predisposed to primary glaucoma when inflammatory or infectious processes develop, resulting in mixed‐risk glaucoma.

### 3.5 Glaucoma and Affected Breeds

The two most common rabbit breeds examined were the Holland Lop (34.2%; 76/222) and the Netherland Dwarf (32.9%; 73/222), each comprising approximately one‐third of the total animals examined (Table 1). All types of glaucoma (i.e., primary, secondary, and mixed‐risk) were represented in these two breeds, but not in the other breeds, possibly due to insufficient number of animals. The prevalence of glaucoma of all types within each breed was 40.8% in the Holland Lop and 20.5% in the Netherland Dwarf, where the former breed had higher prevalence of glaucoma (chi‐square test for independence 2 × 2 contingency table; *p* = 0.051). There was a significant difference between these breeds and all other breeds examined (chi‐square test for independence *m* × *n* contingency table; *p* = 0.05).

### 3.6 Gonioscopy

**FIGURE 2:**
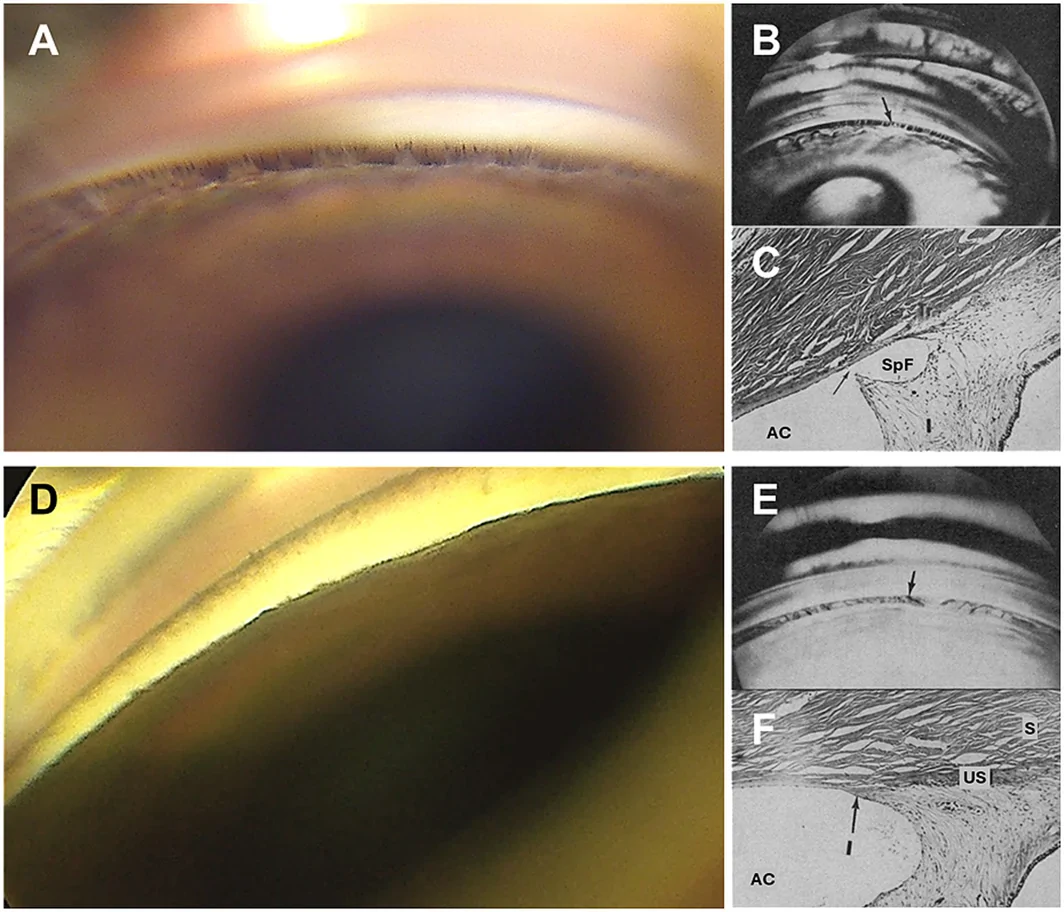
(A) Gonioscopic photograph of an open iridocorneal angle in a normal rabbit in the current study (case #50, right eye). The inferior segment is shown. The pectinate ligaments span a wide space across the trabecular meshwork. Fine pectinate ligaments contacting the cornea appear to become fused toward the iridal side, resulting in broader gaps between blocks of the pectinate ligaments. (B) Goniophotograph of a normal rabbit from a historic publication by Lee. The iridocorneal angle is open, spanned by fine pectinate ligaments (arrow). (C) Histologic section of the same eye as B by Lee, showing the space of Fontana (SpF), pectinate ligaments (arrow), drainage channel (Tr), and normal insertion of iris (I). (D) Gonioscopic photograph of a closed iridocorneal angle in a rabbit affected by glaucoma in the current study (case #14, left eye). The angle is collapsed with no visible pectinate ligaments or trabecular meshwork. (E) Goniophotograph of a glaucomatous rabbit from a historic publication by Lee, showing the filtration angle packed with undifferentiated uveal tissue (arrow). (F) Histologic section of the same eye as E by Lee. The space of Fontana is absent. Abnormal anterior insertion (I) of uveal tissue is apparent. Undifferentiated uveal tissue is closely attached to the sclera and the filtration angle (US). The anterior chamber (AC) and sclera (S) are also indicated (H&E, ×100). Adapted from Ref. [20] with permission.

Of the 33 rabbits diagnosed with primary glaucoma, gonioscopy was performed in 23 (69.7%) animals. Gonioscopy could not be performed in the other 10 cases with primary glaucoma due to the presence of bilateral glaucoma or corneal disease. However, it should be noted that the diagnosis of primary glaucoma could still be determined in these cases based on the lack of intraocular inflammation or underlying ocular conditions as examined using slit lamp and/or ocular ultrasound. Although the anatomy of the iridocorneal angle of rabbits differs from that of canines and felines, we used the angle grading schemes with four categories: open, slightly narrow, narrow, and closed. These categories correspond to the scores 1, 2, 3, and 4 established for canines as described in the OFA gonioscopy form (version 6/24/22). Gonioscopy in normal rabbits showed open angle (Figure 2A), consistent with historical reports (Figure 2B,C). Of the 23 rabbits that were affected by primary glaucoma and had gonioscopy performed, the angle was found to be closed in 16/23 (69.6%; Figure 2D), consistent with historical reports (Figure 2E,F). Other affected animals included 2/23 closed‐to‐narrow (8.7%), 4/23 narrow (17.4%), and 1/23 narrow‐to‐slightly narrow (4.3%) angle. Of the rabbits that had bilateral gonioscopy, findings were symmetrical between the eyes in two animals (one bilaterally closed; one bilaterally narrow), and similar in one animal (narrow‐to‐slightly narrow in the right and narrow in the left eye). None of the animals affected by primary glaucoma had gonioscopy findings spanning slightly normal to normal.

### 3.7 Sex Differences and Age at Diagnosis

In the primary glaucoma group, there were 21 males including six castrated and 12 females including six spayed, while the secondary glaucoma group consisted of 16 males including two castrated and 11 females including two spayed. All three rabbits in the mixed‐risk glaucoma group were male. There was no significant difference in sex distribution between primary, secondary, and mixed‐risk glaucoma types (chi‐square test for independence *m* × *n* contingency table; *p* = 0.380). Among the total 93 glaucomatous eyes of 63 rabbits detected, a significant difference was found with the onset of glaucomatous eyes between primary and secondary glaucomas (chi‐square test for independence *m* × *n* contingency table; *p* = 0.028).

**FIGURE 3:**
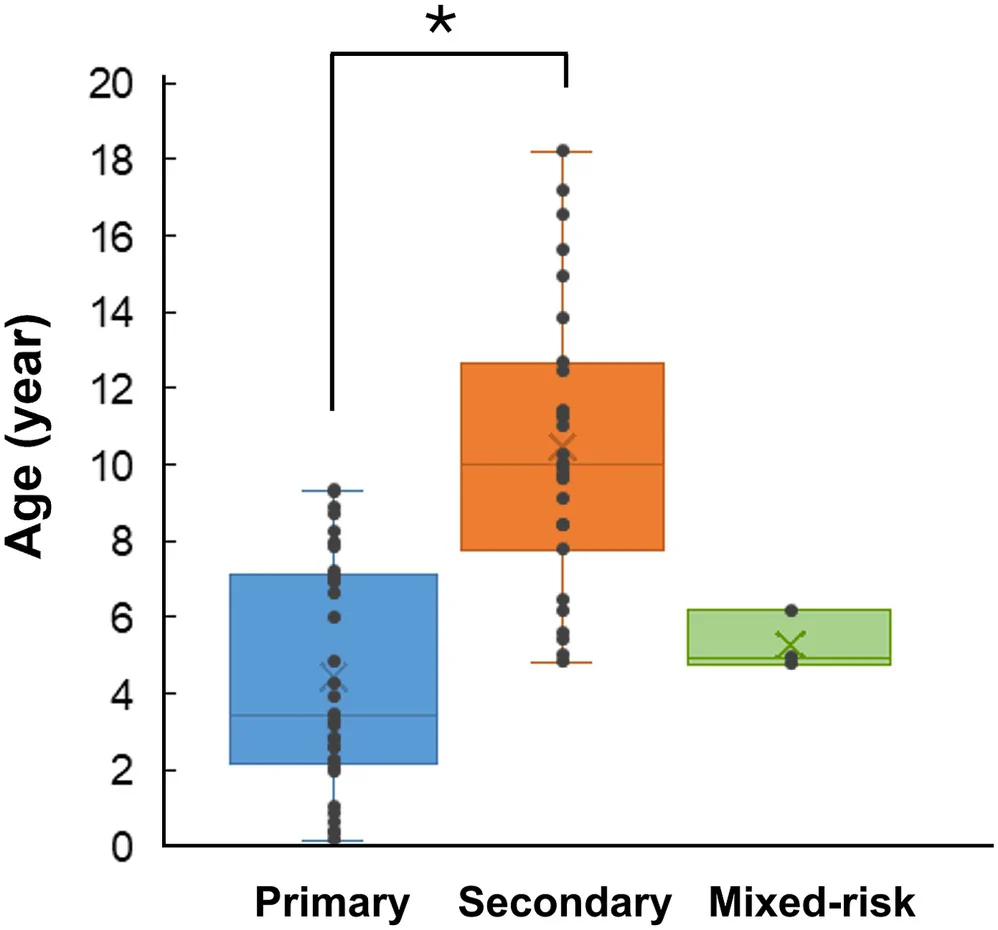
Distribution of the age of onset in primary, secondary, and mixed‐risk glaucomas. Data of individual animals are plotted over the box plots. There was a significant difference in the distribution of the age of onset only between primary and secondary glaucoma groups (*p = 0.000115).

**FIGURE 4:**
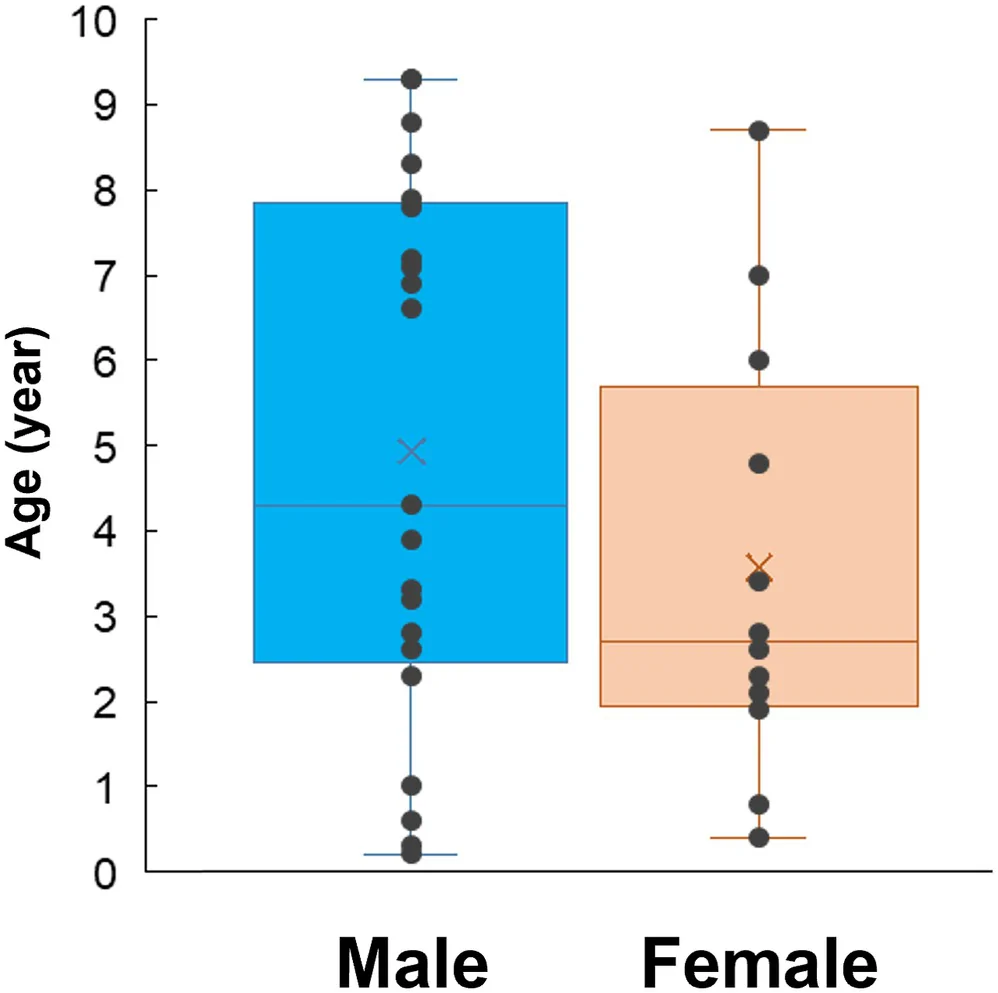
Distribution of the age of onset of primary glaucoma in male (*n* = 21) and female (*n* = 12) rabbits. Data of individual animals are plotted over the box plots. There was increased representation of males at higher ages of disease onset. However, there was no significant difference in the age of onset between the sexes (*p* = 0.2).

**FIGURE 5:**
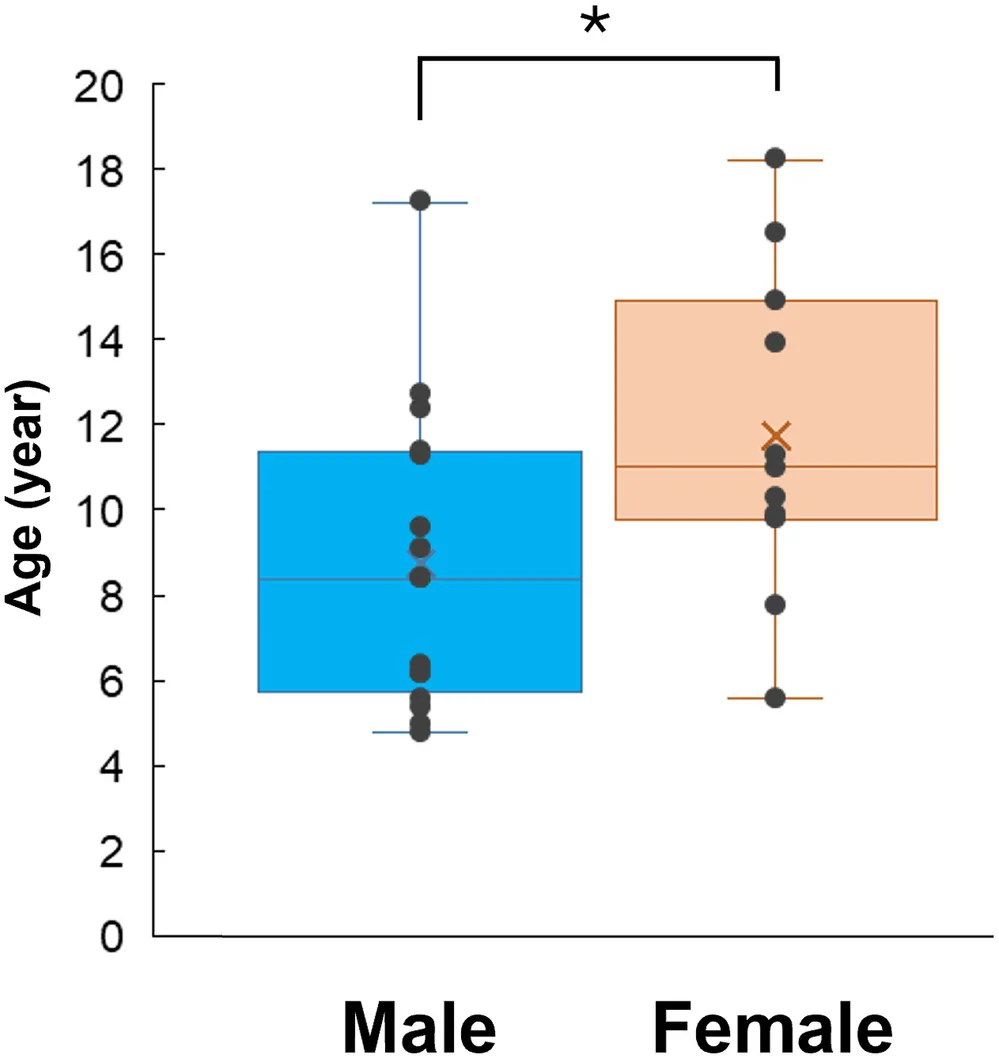
Distribution of the age of onset of secondary glaucoma in male (*n* = 16) and female (*n* = 11) rabbits. Data of individual animals are plotted over the box plots. Females presented with a significantly later age of onset than males (*p = 0.016).

The age at diagnosis of glaucoma was 4.4 ± 2.8 years (range: 0.2–9.3 years) for primary, 10.0 ± 5.3 years (range: 4.8–18.2 years) for secondary, and 5.3 ± 0.8 years (range: 4.8–6.2 years) for mixed‐risk glaucomas (Figure 3). There was a significant difference in the age of onset between the primary and secondary glaucoma groups (*p* < 0.0001). In the simple regression analysis, sex was not identified as a significant predictor of the age of onset in primary glaucoma (*p* = 0.200) (Figure 4). In the simple regression analysis, sex was a significant predictor of the age of onset in secondary glaucoma, where females presented with a significantly later age of onset than males (*p* = 0.016) (Figure 5). Lastly, there was no difference in the laterality of the affected eye (right, left, or both eyes) among the primary, secondary, and mixed‐risk eyes (Kruskal–Wallis test; *p* = 0.320).

### 3.8 Medical Management

A range of 13 topical antiglaucoma medications approved in Japan was used to treat affected rabbits (Table 5). Individual treatment regimens for rabbits with primary, secondary, and mixed‐risk glaucoma are presented in Tables S1–S3.

**TABLE 5:** Ophthalmic drugs used for the management of glaucoma in pet rabbits.

Drug class	Active ingredient(s)	Brand	Approved in the US (year)	Approved in Japan	Notes	Reported rabbit IOP reduction	References
α1‐Adrenergic antagonist	Bunazosin hydrochloride 0.01%	Detantol	No	1985	Increased uveoscleral outflow	Yes	[21]
α2‐Adrenergic agonist	Brimonidine 0.1%	Aiphagan	Yes (1996)	2004	Marketed in the US as Alphagan	Yes	[22]
β‐blocker	Timolol 0.5%	Timoptol	Yes (1978)	1980	—	Yes	[23]
	Levobunolol 0.5%	Mirol	Yes (1985)	2000	Marketed in the US as Betagan	NA	—
β‐blocker/nitric‐oxide donating β‐blocker	Nipradilol 0.25%	Hypadil	No	1990	—	Yes	[24]
Carbonic anhydrase inhibitor + β‐blocker	Dorzolamide 1% + Timolol maleate 0.5%	Cosopt	Yes (1998)	2001	Dorzolamide is marketed primarily as 1% in Japan and exclusively as 2% in the US	NA	[25]
	Brinzolamide 1% + Timolol maleate 0.5%	Azorga	No	2013	Marketed in Europe as Azarga (2008)	Yes	[26]
Rho‐kinase inhibitor	Ripasudil 0.4%	Glanatec	No	2014	—	Yes	[27]
EP2 receptor agonist	Omidenepag isopropyl 0.002%	Eybelis	Yes (2022)	2018	Marketed in the US as Omlonti	Yes	[28]
Prostaglandin‐related (docosanoid)	Unoprostone isopropyl 0.12%	Rescula	Yes (2000)	1994	Marketed in the US as Unoprostone 0.15%	Yes	[29]
Prostaglandin F2α analogues	Latanoprost 0.005%	Xalatan	Yes (1996)	1999	—	Yes^a^	[30, 31, 32]
	Travoprost 0.004%	Travatanz	Yes (2001)	2007	Marketed in the US as Travatan Z	Yes	[33]
	Tafluprost 0.0015%	Tapros	Yes (2012)	2008	Marketed in the US as Zioptan (preservative‐free)	NA^b^	[34]

^a^ Monotherapy did not lower IOP; enhanced IOP‐lowering effect when used in combination with nipradilol.

^b^ Rabbit ocular vascular study supporting ocular hypotensive mechanism.

For primary glaucoma, the most common initial regimen consisted of latanoprost combined with nipradilol (*n* = 7). The median number of medications at baseline was two drugs (mean 1.7). Single‐agent therapy was recorded less frequently and most often involved ripasudil. Treatment modification occurred in the majority of cases during follow‐up. The median number of medications increased to approximately three drugs. The median time to the first treatment adjustment was 17 months (range 1–35 months). Recorded changes included addition of carbonic anhydrase inhibitors and ripasudil. Switching among prostaglandin analogs was also recorded, including transitions from latanoprost to travoprost, tafluprost, or bimatoprost. In most cases, sequential medication changes resulted in multi‐drug regimens during follow‐up.

For secondary glaucoma, initial management varied, and several cases were recorded without topical therapy for cases lacking significantly increased IOP, or those difficult to medicate. The most common initial regimens included carbonic anhydrase inhibitors either alone or in combination, β‐blockers, or nipradilol. The median number of medications at baseline was one to two drugs. Treatment modifications during follow‐up increased the median number of medications to two to three drugs. The time to the first treatment modification ranged from 2 to 13 months. Recorded changes most frequently included addition of ripasudil or fixed carbonic anhydrase inhibitor/β‐blocker combinations. Switching among prostaglandin analogs was also observed in several cases. In some animals, repeated medication adjustments resulted in multi‐drug therapy. In a subset of cases, medical therapy was discontinued following phthisis bulbi or enucleation.

Topical treatments were generally well tolerated. Mild periocular irritation and transient blepharospasm were recorded in a subset of rabbits after administration of dorzolamide‐containing solutions. In these cases, treatment was changed to a brinzolamide‐containing suspensions. No further irritation was recorded after this change, while owners often reported preference of the opaque brinzolamide‐containing suspensions that facilitated visualization of drug instillation over the clear dorzolamide‐containing solutions.

### 3.9 Surgical Management and Histopathology

Vision‐ or globe‐preserving procedures (e.g., laser cyclophotocoagulation, chemical ablation) were not pursued in any of the cases in this retrospective. Surgical intervention was rarely elected largely due to concerns regarding general anesthesia. In addition, affected rabbits typically maintained open eyes with limited overt signs of ocular pain, typical of prey species, further dissuading the owners from electing surgery. Enucleation was reserved for advanced complications, including corneal perforation from buphthalmia and progressive stromal invasion by encapsulated, caseous abscesses associated with *E. cuniculi* infection. During the observation period, enucleation was performed in 3.0% (1/33) of primary glaucoma cases and 14.8% (4/27) of secondary glaucoma cases. Histopathology was available for two cases of secondary glaucoma. In both, *E. cuniculi* infection was suspected based on ocular findings and serology, but clinical signs did not improve with fenbendazole and enrofloxacin. Histopathology confirmed intralenticular spores with associated inflammation and granulomatous changes, consistent with *E. cuniculi* infection.

### 3.10 Long‐Term Evaluation

**FIGURE 6:**
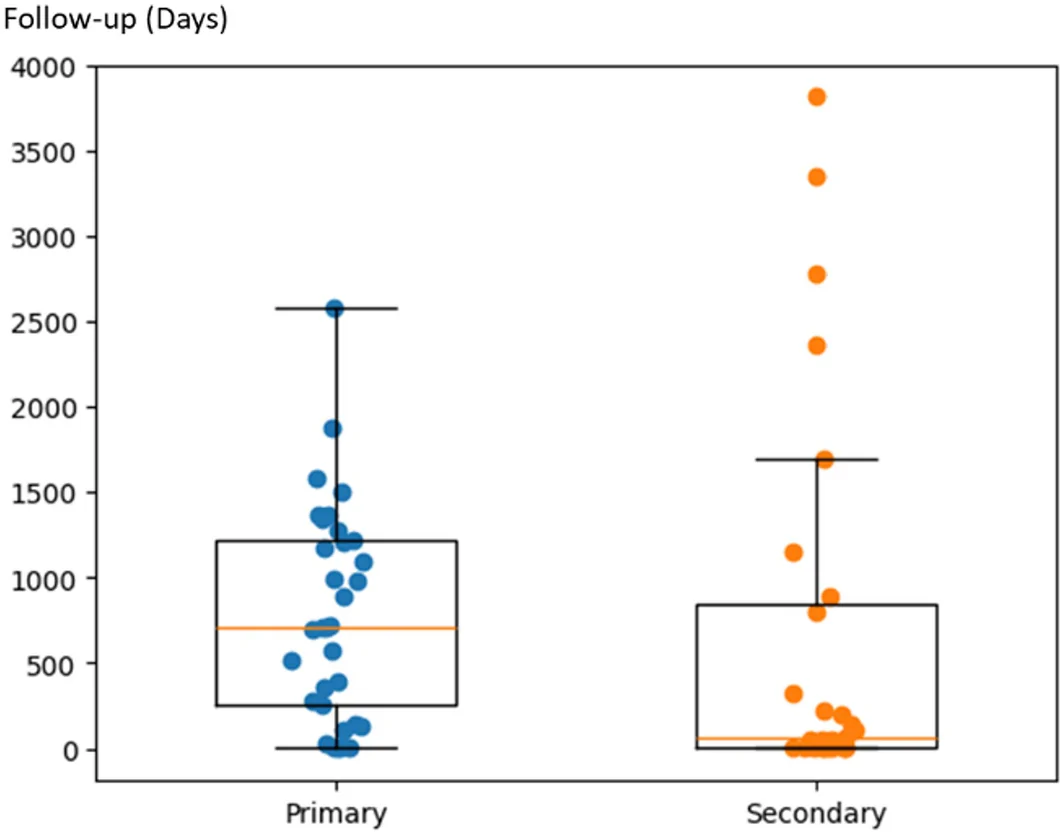
Duration of clinical follow‐up in pet rabbits affected by primary or secondary glaucoma. The *y* axis depicts the days since the initial encounter. Boxes represent the interquartile range (IQR) (25th–75th percentile) with the median shown as a horizontal line. Whiskers extend to 1.5× IQR. Individual points represent each animal, with points beyond the whiskers indicating outliers.

In rabbits undergoing treatment for glaucoma, follow‐up examinations were recommended every 3 months for life when IOP remained controlled, and monthly when uncontrolled, with treatment adjustments made as needed. The median follow‐up duration from initial presentation was 707 days [IQR 126–1299] (mean ± SD, 791 ± 625 days) for primary glaucoma and 59 days [IQR 1–1623] (mean ± SD, 669 ± 1124 days) for secondary glaucoma (Figure 6). The primary glaucoma group had a significantly longer follow‐up period than the secondary glaucoma group (*p* < 0.05). The secondary glaucoma group showed a bimodal distribution, with many cases having short or no follow‐up and a subset with prolonged durations, whereas primary glaucoma demonstrated a more continuous distribution with a longer median follow‐up (Figure 6). In the mixed‐risk glaucoma group, all three cases had long‐term follow‐up ranging from 322 to 2128 days, thus also allowing extended data collection that supported the clinical classification.

## 4 Discussion

Published studies involving client‐owned pet rabbits with glaucoma remain limited, with several reports focusing more on treatment than disease prevalence [15, 16]. Recently, Damstén et al. evaluated client‐owned rabbits in France and reported a glaucoma prevalence of 16.6% (35/211) [17]. Primary glaucoma in that population was suspected in only 11.4% (4/35) of glaucomatous rabbits. In contrast, the prevalence of glaucoma in the present Japanese cohort was higher at 28.4% (63/222), with primary glaucoma accounting for 52.4% (33/63) of affected rabbits. However, the comparison should be interpreted with caution, as the French cohort in the Damstén study had a smaller glaucomatous sample size (*n* = 35) than the Japanese cohort in the current study (*n* = 63). While both studies included cases presented for ophthalmic examination and may therefore be biased toward ocular disease, the higher rate of glaucoma diagnosis and the greater proportion of primary glaucoma may reflect differences in genetic predisposition among rabbit populations.

Studies in laboratory New Zealand White rabbits have indicated that a form of hereditary glaucoma in rabbits most likely is autosomal recessive with incomplete penetrance (semi‐lethal) [7]. In these rabbits, glaucoma typically manifests in three to six months of age and is associated with variable IOP elevation, corneal clouding and edema, and globe enlargement, consistent with congenital glaucoma [6, 7, 8, 13, 35]. For example, Kolker et al. diagnosed glaucoma when the corneal diameter exceeded 17 mm and the IOP increased from 5 months of age [8]. In the present study, the age at onset of primary, secondary, and mixed‐risk glaucoma was 4.4 ± 2.8 years (range: 0.2–9.3 years), 10.0 ± 5.3 years (range: 4.8–18.2 years), and 5.3 ± 0.8 years (range: 4.8–6.2 years), respectively (Figure 3). These pet rabbits with primary glaucoma were notably older at the onset of glaucoma compared to the New Zealand White rabbits in laboratory‐based studies indicating a distinct genetic etiology [6, 8, 13, 35]. The average lifespan of pet rabbits is 7–10 years or less [36]; thus, the onset of primary glaucoma at 4.4 ± 2.8 years of age as identified in the current study is considered to occur at middle age, comparable to the typical age of onset of primary glaucoma in canines.

In a clinical and pathological study, gonioscopic, histopathologic and ultrastructural examinations suggested abnormal iridocorneal angle and pectinate ligament dysplasia as the underlying mechanisms of hereditary glaucoma in New Zealand White rabbits [37]. While gonioscopy in rabbits [20] can be technically challenging, alternative imaging modalities have been explored. Li Puma et al. compared the evaluation of the iridocorneal angle using gonioscopy, SD‐OCT, high‐resolution ultrasound, and Scheimpflug imaging (Pentacam HR) in pet rabbits [18]. They concluded that the gonioscopy grade correlated well with the angle‐recess area based on SD‐OCT and recommended SD‐OCT as a noncontact method for evaluating the angle in companion rabbits. In the current study, gonioscopy was performed by a single board‐certified veterinary ophthalmologist (KK) experienced in gonioscopy and research expertise in goniodysgenesis in glaucomatous dogs [38, 39].

To the authors' knowledge, reports that investigated pet rabbits with glaucoma are limited [15, 16, 17]. In one study, glaucoma was diagnosed based on clinical signs combined with IOP, and gonioscopy was performed on three contralateral (unaffected) eyes, and their results revealed one narrow and two narrow‐to‐closed angles [15]. It was suspected that the prevalence of primary glaucoma among glaucoma cases in that study was 27.3% (3/11). If gonioscopy had been performed in the remaining cases, the number of eyes with goniodysgenesis might have been higher. In our present study, cases of all glaucoma types accounted for 28.4% (63/222) of pet rabbits examined, of which primary glaucoma was most prevalent, accounting for 52.4% (33/63) of glaucoma cases. This suggests that primary glaucomatous disease may be more common in pet rabbits than previously recognized. These findings differ from an earlier report with a lower prevalence of primary glaucoma.

Primary glaucoma showed no sex difference in age of onset, whereas secondary glaucoma developed significantly later in females (*p* = 0.016). The reason for this sex difference is unclear but may reflect differences in susceptibility to underlying etiologies. For example, 9 of 11 rabbits with *E. cuniculi*‐associated secondary glaucoma were male. In addition, all three mixed‐risk glaucoma cases were male and had secondary glaucoma associated with *E. cuniculi*. Further investigation in a larger rabbit population is warranted to clarify sex differences according to etiology.

Clinically, in the authors' experience, phthisis bulbi seemed to develop less frequently in rabbits with glaucoma than in dogs. Differences in ocular biomechanical properties between species may contribute to this observation, although larger multicenter studies with long‐term follow‐up are needed to confirm this impression. Corneal perforation was recorded in one case of primary glaucoma and four cases of secondary glaucoma. In these cases, rupture was most commonly associated with exposure keratitis secondary to buphthalmia or invasion of the corneal stroma by encapsulated intraocular abscesses associated with *E. cuniculi* infection. Cases diagnosed with glaucoma are recommended to undergo regular follow‐up examinations to monitor IOP and adjust medical therapy. The relatively high rate of repeat follow‐up visits observed in the present study reflects the need for long‐term monitoring and medical management, particularly in rabbits with primary glaucoma. A greater proportion of rabbits with secondary glaucoma were lost to follow up after the initial examination. This pattern may suggest that secondary glaucoma can follow a more severe clinical course despite treatment. Consequently, a larger proportion of secondary glaucoma cases were classified based on a single examination, although diagnosis was generally more straightforward than for primary glaucoma, which required exclusion of underlying causes.

In this report, we define “mixed‐risk glaucoma” as a clinical category in which one eye presents with secondary glaucoma while the fellow eye is gonioscopically at risk for primary glaucoma. It is important to recognize that rabbits presenting with unilateral secondary glaucoma may still have been predisposed to primary glaucoma, and the contralateral clinically unaffected eye could benefit from prophylactic treatment. Whether an underlying risk for primary glaucoma influences the development of secondary glaucoma remains uncertain. Only three cases in this study met the criteria for mixed‐risk glaucoma, highlighting the need for further investigation in larger cohorts. Notably, in the present study, gonioscopy was not routinely performed in the fellow eye of rabbits presenting with secondary glaucoma. However, in light of our findings of mixed‐risk glaucoma cases, we recommend gonioscopy in the eye(s) in which the examination is feasible in both primary and secondary glaucoma presentations.

Elevated IOP, although not always present in human glaucoma patients, is a major risk factor for glaucoma‐associated optic neuropathy and is the main focus of medical therapy. In the present retrospective cohort, 13 different topical antiglaucoma medications were used either as monotherapy or in combination. The safety and efficacy of many of these drugs were previously evaluated in laboratory rabbits, though more recently, drug testing in rabbits seems to be optional over smaller rodents and non‐human primates, resulting in limited applicable data for their use in pet rabbits. Four of the medications used in this study are approved in Japan but not in the United States, providing additional treatment options for Japanese clinicians. One such drug is ripasudil (Glanatec), a Rho kinase (ROCK) inhibitor shown to reduce IOP in rabbits [27]. Since its introduction in 2014, ripasudil was frequently used to manage both primary and secondary glaucoma, suggesting potential utility in glaucoma management in pet rabbits. Latanoprost, a prostaglandin F2α analogue widely used for canine glaucoma, has limited IOP‐lowering efficacy as monotherapy in rabbits but is effective when combined with nipradilol [31]. Accordingly, latanoprost was typically used in combination with nipradilol in the present study. Treatment modifications during follow‐up generally included increased dosing frequency, switching between drug classes, and addition of multiple topical agents. Because medication availability and clinician preference evolved during the study period, prospective studies are needed to better evaluate long‐term treatment efficacy in pet rabbits.

Although previous reports describe favorable outcomes with surgical management of medically refractory glaucoma in pet rabbits, including enucleation and chemical ablation [15], surgical interventions were rarely elected in the present cohort. In veterinary ophthalmology, enucleation is commonly recommended for blind, painful eyes with uncontrolled IOP. However, based on the authors' clinical experience, pet owners in Japan often express reluctance toward surgical interventions, particularly visually conspicuous procedures such as enucleation. Owners frequently prefer prolonged medical management, which may explain the predominance of long‐term medical therapy observed in this cohort.

Although the *bu* locus associated with buphthalmos has been recognized since the 1960s, the specific genetic variant and chromosomal location remain unknown. To the authors' knowledge, no genetic test currently exists for screening rabbits for glaucoma‐associated variants. The present study therefore provides foundational clinical data for ongoing genetic investigations aimed at identifying variants associated with glaucoma in rabbits for a particular breed or segregating across multiple breeds. Laboratory *bu*/*bu* rabbits develop progressive IOP elevation at 1–3 months postnatally, resembling congenital glaucoma [10]. In contrast, the age of onset in the present pet rabbit population was 4.4 ± 2.8 years, consistent with non‐congenital primary glaucoma. This suggests that the *bu* locus may differ from the genetic basis of primary glaucoma in pet rabbits, although modifier genes or genomic background could influence disease expression. Ongoing genomic studies in these rabbits are expected to clarify the role of the *bu* locus or identify additional loci involved in disease susceptibility.

Yuschenknoff et al. reported that the average age of onset of glaucoma diagnosed in the rabbits was eight years of age, ranging from eight months to 12 years, although the types of glaucoma were not differentiated [15]. Limited histopathologic and gonioscopic findings suggested that some may be affected by a disease process similar to primary closed‐angle glaucoma in adult canines rather than the buphthalmic trait (*bu*) in New Zealand White rabbits. In our current study, Holland Lop and Netherland Dwarf breeds comprised 67.1% (149/222) of the population examined, where the prevalence of glaucoma among ophthalmic patients in these breeds was 40.8% (31/76) and 20.5% (15/73), respectively. These relatively high prevalences suggest a potential genetic predisposition in these breeds. Therefore, genomic studies are warranted to identify the variants associated with the *bu* locus or other loci contributing to primary glaucoma in pet rabbits, particularly in Holland Lop and Netherland Dwarf breeds.

This retrospective study has several limitations. The observed glaucoma prevalence of 28.4% (63/222) could reflect referral bias examined by a board‐certified ophthalmologist and may overrepresent the true prevalence in the general pet rabbit population. Another limitation is the limited understanding of the relationship between gonioscopic findings and glaucoma development in rabbits, as well as the distribution of angle morphology in normal rabbits. Prospective studies evaluating angle structure in unaffected rabbits along with longitudinal IOP monitoring would provide important context. Misclassification of glaucoma subtypes remains possible, particularly in cases of possible subclinical uveitis or lack of gonioscopy data, limit diagnostic certainty. However, the higher rate of bilateral disease in primary glaucoma and the availability of long‐term follow‐up support the subtypes determined for each rabbit. Future studies should incorporate standardized diagnostic protocols including gonioscopy, *E. cuniculi* testing, and histopathologic evaluation of enucleated eyes.

In conclusion, both primary and secondary glaucomas are prevalent among pet rabbits in Japan. The age of onset is earlier in primary glaucoma (4.4 ± 2.8 years) than in secondary glaucoma (10.0 ± 5.3 years). While there were no significant sex differences in the age of onset for primary glaucoma, females had significantly later onset for secondary glaucoma. Gonioscopy revealed closed iridocorneal angle in 69.6% (16/23) of tested animals affected by primary glaucoma. The most common underlying ocular condition leading to secondary glaucoma was suspected to be *E. cuniculi* infection. Research into the genetic basis of glaucoma in rabbits, especially Holland Lop and Netherland Dwarf breeds, is warranted.

## Author Contributions

**Yasutsugu Miwa:** supervision. **Makoto Nakata:** project administration. **Keiko Miyadera:** writing – review and editing, data curation, investigation, visualization, validation, formal analysis, supervision, writing – original draft. **Kumiko Kato:** conceptualization, visualization, writing – original draft, formal analysis, data curation, investigation, methodology, supervision, project administration.

## Disclosure

AI statement: The authors have not used AI to generate any part of the manuscript.

## Ethics Statement

This study was approved by the hospital boards of the Japan Exotic Animal Medical Center and Kumi Animal Hospital. Animal owners or owner representatives provided written informed consent for enrollment in the study, the procedures and therapy undertaken, and the publication of data and images.

## Conflicts of Interest

The authors declare no conflicts of interest.

## Supporting information


**Table S1:** Changes in anti‐glaucoma treatment regimen and IOP in rabbits diagnosed with primary glaucoma.
**Table S2:** Changes in anti‐glaucoma treatment regimen and IOP in rabbits diagnosed with secondary glaucoma.
**Table S3:** Changes in anti‐glaucoma treatment regimen and IOP in rabbits diagnosed with mixed‐risk glaucoma.

## Data Availability

The data that support the findings of this study are available from the corresponding author upon reasonable request.
